# Targeting Cartilage Degradation in Osteoarthritis

**DOI:** 10.3390/ph14020126

**Published:** 2021-02-05

**Authors:** Oliver McClurg, Ryan Tinson, Linda Troeberg

**Affiliations:** Norwich Medical School, University of East Anglia, Bob Champion Research and Education Building, Rosalind Franklin Road, Norwich NR4 7UQ, UK; O.Mcclurg@uea.ac.uk (O.M.); rtinson@googlemail.com (R.T.)

**Keywords:** osteoarthritis, cartilage, metalloproteinase, targeting

## Abstract

Osteoarthritis is a common, degenerative joint disease with significant socio-economic impact worldwide. There are currently no disease-modifying drugs available to treat the disease, making this an important area of pharmaceutical research. In this review, we assessed approaches being explored to directly inhibit metalloproteinase-mediated cartilage degradation and to counteract cartilage damage by promoting growth factor-driven repair. Metalloproteinase-blocking antibodies are discussed, along with recent clinical trials on FGF18 and Wnt pathway inhibitors. We also considered dendrimer-based approaches being developed to deliver and retain such therapeutics in the joint environment. These may reduce systemic side effects while improving local half-life and concentration. Development of such targeted anabolic therapies would be of great benefit in the osteoarthritis field.

## 1. Introduction

Osteoarthritis (OA) is a degenerative joint disorder that affects over 250 million people worldwide [1,2], causing pain, stiffness, and impaired movement. OA most commonly occurs in the knee, hip, and hand joints [2,3], but can also affect other articulating joints. The disease is characterized by progressive loss of cartilage, which impairs smooth articulation of opposing bones in the joint, with remodeling of subchondral bone (including sclerosis and osteophyte formation) and other joint tissues (such as the meniscus, ligaments, synovium, and intrapatellar fat pad) also contributing to joint degeneration [4,5,6]. 

There are currently no disease-modifying OA drugs (DMOADs) available, with treatment limited to analgesia for early-stage disease and surgical joint replacement for late-stage disease. The development of effective drugs to treat OA is thus of utmost importance. Much research in this area is focused on cartilage, and aims to identify approaches for either stopping cartilage breakdown or promoting cartilage repair [2,7].

Cartilage consists of a single cell type, the chondrocytes, which synthesize and are embedded in a relatively large volume of extracellular matrix (ECM) that is critical for the tissue’s mechanical properties [8]. The two most abundant components of the cartilage ECM are type II collagen and aggrecan, which confer tensile strength and resistance to compression, respectively. In healthy cartilage, anabolic synthesis of ECM components is balanced with their catabolic turnover to maintain joint homeostasis. In OA, changes in the mechanical environment of the joint (e.g., by injury or ageing) shift this balance towards degradation, with breakdown of type II collagen and aggrecan by metalloproteinases leading to progressive joint damage [9,10]. Expression of several of these metalloproteinases, as well as pro-inflammatory mediators, is induced by mechanical destabilization of the joint [9]. Additional factors including chondrocyte senescence [11], oxidative stress [12,13,14,15], and/or inflammation can also increase metalloproteinase expression and tip the balance towards cartilage breakdown.

Collagenases such as matrix metalloproteinase 13 (MMP-13) are thought to be important for degradation of type II collagen in OA [16,17,18], while aggrecanases such as adamalysin with thrombospondin motifs 5 (ADAMTS-5) drive aggrecan loss [19,20]. Activity of these metalloproteinases is increased in OA through a variety of molecular mechanisms, including increased expression [16,21], decreased endocytic clearance [22,23], and a reduction in levels of their endogenous inhibitor, tissue inhibitor of metalloproteinases 3 (TIMP-3) [24]. These enzymes have been the target of multiple academic and industry DMOAD programs, but this approach has been challenging, due to high homology with several homologous metalloproteinases that have important homeostatic roles in processes such as wound healing and cell migration. 

An alternative approach to OA therapy is to boost the intrinsic repair capacity of cartilage, through delivery of growth factors that can promote ECM synthesis and cartilage repair. For example, growth factors in the fibroblast growth factor (FGF), transforming growth factor β (TGF-β), and Wnt families are known to have important roles in cartilage development, and can also promote repair of damaged adult cartilage. However, development of growth factor-based DMOADs has also been challenging, due to complexities in their signaling pathways and unexpected effects including fibrosis and osteophyte formation [25]. 

Here we provided an update on recent developments and clinical trials in the search for metalloproteinase- and growth factor-focused DMOADs, as well as approaches being explored for delivery and retention of potential OA therapeutics in the joint. Such delivery strategies are increasingly seen as a promising way to limit effects of potential DMOADs to the joint and thus to reduce adverse systemic effects.

## 2. Novel Strategies for Inhibiting Cartilage Breakdown

### 2.1. Inhibiting ADAMTS Activity

The role of MMPs and ADAMTSs in OA cartilage degradation is well appreciated, making these enzymes attractive targets for DMOAD development [26,27,28,29,30]. ADAMTS-5 has received particular attention [26,27,28], because reversible degradation of aggrecan is thought to precede irreversible collagen loss [31,32], and ADAMTS-5 is thought to be the critical “aggrecanase” in both murine and human OA [19,20,33]. 

As with MMP inhibitors before them [34,35], design of ADAMTS-5 inhibitors has been challenging, with high homology between metalloproteinase active sites leading to off-target inhibition of metalloproteinases with homeostatic functions in processes such as angiogenesis, cell migration, and wound healing [26,27,28]. Strategies to improve selectivity include development of bi-specific, cross-domain antibody scaffolds. This approach has been utilized by Merck Serono, whose bi-specific nanobody against ADAMTS-5 (M6495) has been shown to protect against surgically-induced murine OA in vivo [36]. M6495 was recently out-licensed to Novartis, and phase II trials are expected. Targeting exosites outside of the metalloproteinase active site is another promising approach, recently shown to enable development of an inhibitor with high selectivity for ADAMTS-5 over ADAMTS-4 [37]. 

In addition to adverse off-target effects, metalloproteinase inhibitors can also potentially have unwanted on-target effects, arising from expression of target aggrecanases and collagenases outside of the joint. For example, expression of ADAMTS-5 in the vasculature and heart valves is thought to underlie cardiovascular side-effects observed with the anti-ADAMTS-5 inhibitory antibody GSK2394002 [38]. Potential roles of ADAMTS-5 in wound healing, glucose metabolism, inflammation, and neural plasticity (reviewed in [28]) warrant further investigation. Similarly, MMP-13 has physiological roles in wound healing [39], muscle regeneration [40], and fracture healing [41,42] that should be kept in mind when designing MMP-13 inhibitors for OA therapy. 

Strategies other than direct inhibition of metalloproteinase activity are also being explored. For example, siRNAs against *Adamts5* [43] and *Mmp13* [43,44] have both been successfully used to reduce cartilage degradation in rodent OA models. ADAMTS-5 and MMP-13 are also post-translationally regulated by endocytosis and lysosomal degradation, mediated by the low-density lipoprotein receptor-related protein 1 (LRP1) scavenger receptor [22,23,45], and this protective endocytic process is impaired in OA cartilage [22,23,46]. Antibodies inhibiting shedding of LRP1 were found to inhibit cartilage degradation in vitro [46]. 

### 2.2. Augmenting Levels of Endogenous TIMPs

Another potential approach is to enhance levels of the endogenous inhibitors of MMPs and ADAMTSs in cartilage. Among the 4 mammalian tissue inhibitors of metalloproteinases (TIMPs), TIMP-3 is the only one that effectively inhibits both MMPs and ADAMTSs [47], with TIMP-1, TIMP-2, and TIMP-4 having more restricted inhibitory profiles and largely targeting MMPs (reviewed in [48]). Although there is no significant change in mRNA expression of TIMP-3 in OA cartilage [24,49], levels of the protein itself are reduced [24]. Subsequent studies in our group showed that, as with ADAMTS-5 and MMP-13, TIMP-3 levels are also post-translationally regulated by LRP1-mediated endocytosis [50]. It is not currently clear how endocytosis of metalloproteinases and TIMP-3 is coordinated in cartilage, although factors such as their differential affinity for extracellular matrix glycosaminoglycans and localization in tissue are likely to be key [51], seeing as their affinity for LRP1 is not markedly different [52]. Mutants of TIMP-3 with reduced affinity for LRP1 were found to have longer half-lives in cartilage and improved chondroprotective activity [53]. Sulfated glycans and glycan mimetics that block TIMP-3 binding to LRP1 were found to be similarly protective in cartilage explant assays [54,55] and murine OA models [56], raising the possibility that small molecule inhibitors of TIMP-3 binding to LRP1 may inhibit cartilage matrix degradation. TIMP-3-directed approaches would have to overcome the same issues of specificity and joint targeting as discussed for metalloproteinase-directed approaches. 

## 3. Promoting Cartilage Repair with Anabolic Growth Factors

An alternative approach to directly targeting metalloproteinases is to stimulate cartilage repair through delivery of growth factors that inhibit metalloproteinase-mediated cartilage degradation while promoting anabolic repair pathways (reviewed by [57]). Here we discuss progress and recent or current clinical trials in this area. 

### 3.1. Platelet-Rich Plasma Therapy

Platelet-rich plasma (PRP) therapy for OA involves intra-articular injection with a preparation of autologous plasma containing high platelet levels. Once in the joint, platelets are activated by abundant cartilage ECM proteins, leading to release of their cytoplasmic components, including several anabolic growth factors such as TGF-β1, platelet-derived growth factor (PDGF), insulin-like growth factor (IGF), and FGF2, which promote aggrecan and collagen synthesis, while reducing expression and activity of catabolic metalloproteases [58].

In vitro studies on the effects of PRP on chondrocyte gene expression have yielded mixed results. For example, PRP prepared from porcine blood was found to have anabolic effects on porcine chondrocytes cultured in alginate beads, stimulating an increase in DNA, glycosaminoglycan, and type II collagen content relative to cells cultured with platelet-poor plasma or 10% fetal bovine serum [59]. Similarly, PRP has been shown to increase expression of *COL2A1* [60,61] and *ACAN* [60,61] in human OA chondrocytes, while decreasing interleukin-1β (IL-1β)-induced *ADAMTS4* expression [60]. However, other studies have found PRP to have no beneficial effects on *COL2A1* expression in human OA chondrocytes [62], or have reported adverse effects such as dedifferentiation of chondrocytes to a fibroblast-like phenotype [63]. The reason for these discrepancies is not clear, but may result from differences in the method of PRP preparation. For example, comparison of two commercially available kits showed that white blood cell levels were higher in some preparations than others, with increased neutrophil levels correlating with higher concentrations of pro-inflammatory cytokines such as IL-1β [64], which may promote MMP activation and cartilage breakdown rather than repair.

Clinical trials of PRP therapy have also shown mixed results, although comparison between randomized control trials (RCT) trials of PRP are complicated by variation in their control arms, with some comparing PRP to saline [65], and others comparing it with hyaluronan (HA) [66,67,68]. Among studies that compared PRP with HA, both positive and negative results have been reported. For example, PRP has been shown to reduce Western Ontario and McMaster Universities Osteoarthritis Index (WOMAC) scores more significantly than HA for up to 6 months [66] and to improve self-reported pain more effectively than HA for up to 12 months [67], while other studies have found PRP to be no more effective than HA at improving various patient-reported outcome measures at 2, 6, and 12 months after treatment [68]. As with in vitro studies of PRP, these different RCT outcomes may reflect differences in PRP preparation, with double filtration of plasma more likely to remove leukocytes than centrifugation [69]. 

RCTs of PRP have largely assessed patient-reported outcome measures, and there is relatively little information on whether PRP can alter structural joint outcomes. A recently completed but as yet unpublished RCT (NCT03491761) compared the effects of PRP and HA on knee OA by measuring objective changes such as cartilage thickness in addition to subjective measures such as WOMAC scoring. Such studies are likely to shed light on whether PRP can directly promote cartilage repair and regeneration in vivo. An additional challenge in this area is the requirement for cheap and efficient methods of producing PRP fractions with low leukocyte levels. 

### 3.2. FGF18 Promotes Cartilage Anabolism

While FGF2 has been shown to have both anabolic and catabolic effects on chondrocytes (see Section 3.5, Considerations around Receptor Expression and Downstream Signaling), the current literature supports a chondroprotective, anabolic role for FGF18. For example, in vitro studies showed that FGF18 increased proliferation and proteoglycan production by primary human and porcine chondrocytes [70], and in vivo studies have demonstrated that intra-articular injection of FGF18 significantly reduced cartilage degeneration in rat pre-clinical OA models [71,72].

Following on from these promising studies, pharmaceutical interest in FGF18 has grown. Nordic Biosciences and Merck/EMD Serano developed a truncated form of FGF18, named sprifermin, which lacks the signal peptide and 11 C-terminal amino acids [73]. This modified form of FGF18 retains biological activity, with sprifermin shown to dose-dependently stimulate proliferation of cultured human and porcine chondrocytes, and to increase glycosaminoglycan and type II collagen accumulation, while decreasing *ADAMTS5* expression [73]. 

A number of human clinical trials on sprifermin have now been completed, with intra-articular injection of 3–300 μg of the growth factor followed by evaluation of joint structural parameters and patient-reported outcome measures for up to 2 years [74,75,76].

Dahlberg et al. conducted a first-in-human double-blind RCT with dose escalation that established safety in humans [74]. Overall, treatment-emergent adverse events were not increased in the sprifermin-treated cohort compared to placebo. Twice as many acute inflammatory reactions were seen in the sprifermin-treated cohort (12.7%) as in the placebo group (5.5%), but overall sprifermin was deemed tolerable. The study found no difference in Mankin scores, joint space width or semi-quantitative MRI parameters between the cohorts, although the study was not sufficiently powered for such comparisons with only 55 participants [74].

A second dose-escalating RCT failed to meet its primary endpoint, with no significant change in the thickness of cartilage in the central medial femorotibial compartment detected by quantitative magnetic resonance imaging (MRI) at 6 and 12 months [75]. However, sprifermin did cause a statistically significant reduction in the loss of total and lateral femorotibial cartilage thickness and in joint space width narrowing in the lateral femorotibial compartment compared with the placebo group. WOMAC pain scores improved significantly in both the treatment and control arms, although the improvement was significantly less in the sprifermin-treated cohort than in the placebo group [75]. 

Finally, the FORWARD phase II RCT completed in 2019 reported statistically significant and dose-dependent increases in total femorotibial cartilage thickness after 2 years in cohorts treated with 100 µg of sprifermin every 6 or 12 months compared to placebo [76]. Furthermore, significant increases in cartilage thickness were observed in both the medial and lateral femorotibial compartments, along with significant dose-dependent effects on joint space width in the lateral, but not medial, compartment in cohorts treated with 100 µg of sprifermin. Lower doses of sprifermin did not cause significant improvements. As in the previous sprifermin RCT [75], there was no statistically significant change in WOMAC pain scores between treatment cohorts, with analgesia use similar across treatment groups [76]. There was also no significant improvement in function or stiffness sub-scores. 

Taken together, these RCTs support sprifermin having a chondroprotective effect, with positive effects on cartilage thickness and joint space narrowing. Effects on pain and stiffness have not been demonstrated, reflecting the broader question of whether OA pain correlates with structural joint changes (see Section 5, Broader Challenges in DMOAD Development).

### 3.3. Wnt Pathway Inhibition

Wnt signaling is required for cartilage and bone development and homeostasis, but sustained or elevated Wnt signaling in chondrocytes promotes their proliferation and hypertrophic differentiation, with deleterious effects on cartilage homeostasis (reviewed in [77,78,79]). Inhibition of Wnt signaling is thus being explored as a potential therapy for OA. This approach could have the added benefit of inducing differentiation of mesenchymal stem cells (MSCs) into chondrocytes, since Wnt signaling in MSCs promotes their differentiation into osteoblasts rather than chondrocytes [80]. MSCs are present in elevated numbers in the synovial fluid of OA patients [81], suggesting that while OA joints have the potential to repair, the osteoarthritic environment does not support differentiation of resident MSCs into cartilage-forming chondrocytes. Inhibitors of Wnt signaling may thus both inhibit cartilage degradation and harness its potential for repair. 

SM04690 (Lorecivivint, Samumed), a small molecule inhibitor of Wnt signaling, has progressed from in vitro assessment to human clinical trials. In vitro, SM04690 inhibited Wnt pathway activation and induced differentiation of human MSCs into chondrocytes while inhibiting expression of catabolic metalloproteinases [82,83]. In a rat anterior cruciate ligament transection (ACLT) model of OA, intra-articular injection of SM04690 a week after surgery significantly reduced cartilage degradation 12 weeks later [83]. The mechanism of action is proposed to be through inhibition of the intranuclear kinases CDC-like kinase 2 (CLK2) and dual-specificity tyrosine phosphorylation-regulated kinase 1A (DYRK1A), without affecting β-catenin [84].

A subsequent phase I RCT showed that SM04690 is well tolerated and safe in humans [85]. Participants with moderate to severe OA received a single intra-articular dose of SM04690 (*n* = 48 treated with 0.03 to 0.23 mg SM04690, compared with *n* = 11 in the placebo arm), with safety and efficacy evaluated 24 weeks later. Adverse effects potentially related to treatment were observed primarily in those receiving the highest dose of SM04690 (0.23 mg), and included arthralgia, joint swelling and stiffness (in 5 subjects), as well as gastrointestinal effects (in 4 subjects) [85]. 

In a phase IIa clinical trial of SM04960 completed in 2018, clinically meaningful improvements in WOMAC pain and function scores were seen in all groups, including the placebo group who received an intra-articular injection of PBS [86]. The trial thus did not meet its primary endpoint of a significant reduction in the WOMAC pain score compared with placebo 13 weeks after a single intra-articular dose of SM04960. However, WOMAC pain scores were significantly lower at the 52-week time point in the group treated with 0.07 mg of SM0496 than in the placebo group [86]. Medial joint space width was also significantly improved at 52 weeks in this treatment group compared with placebo [86]. 

The Wnt inhibitors SAH-Bcl9 and StAx-35R are also currently under early investigation in OA [87]. These compounds have been shown to inhibit Wnt signaling in cancers, and also inhibited Wnt3a-induced downregulation of chondrogenic markers such as *COL2A1* and *SOX9* in human OA cartilage explants in vitro [87]. 

Effective and sustained therapeutic targeting of this pathway is likely to be challenging, with multiple ligands and antagonists influencing both canonical and non-canonical Wnt signaling (reviewed in [78,79]), and potential reciprocal antagonism between these pathways shaping the effect on the chondrocyte phenotype [88]. Further investigation of these complexities is required to understand the consequences of Wnt modulation in vivo. 

### 3.4. TGF-β1 Supplementation 

TGF-β1 is the most extensively studied growth factor in cartilage, with in vivo evaluation of its anabolic effects stretching back 3 decades (reviewed in [25]). Genetic evidence for a chondroprotective role for TGF-β1 is strong, with a number of mutations in its signaling cascade associated with increased OA risk [25]. For example, the D-14 polymorphism in asporin, which reduces TGF-β signaling, has been found to increase OA susceptibility in some populations [89,90], and a *SMAD3* single nucleotide polymorphism (SNP) has been linked to increased hip and knee OA in a European 527-strong cohort [91]. These findings are supported by in vivo studies, which showed, for example, accelerated OA in mice that overexpress a dominant negative form of TGF-β receptor II (*Tgfbr2*) [92] or that lack *Tgfbr2* [93] or *Smad3* [94]. However, in addition to its beneficial effects on cartilage, the anabolic effects of TGF-β1 are undesirable in other joint tissues, leading to the synovial fibrosis and osteophyte formation reported in a number of studies (reviewed in [25]). 

Mont and colleagues have conducted a number of clinical trials to assess treatment of knee OA by intra-articular injection of allogeneic chondrocytes transduced to express TGF-β1. A small phase I trial in 12 patients concluded the treatment was safe, and reported improved range of motion and pain scores after 6 and 12 months [95]. Subsequent phase IIa and II RCTs indicated improvements in function and pain [96,97]. Fibrosis and osteophyte formation were not observed, which the authors ascribed to the localized and controlled expression of TGF-β1 achieved by the cell-mediated delivery procedure [97]. Adverse effects such as itching, warm sensations, and knee effusion were limited to the injection site and resolved within a few days [96]. A rat model of allogenic chondrocyte implantation detected infiltrating immune cells in deep but not superficial zones of cartilage [98], suggesting that further studies are warranted to investigate immune responses to allogenic chondrocytes injected into late-stage OA joints. 

A more recent double-blinded, placebo-controlled randomized phase II clinical trial in 102 patients (*n* = 67 in treatment arm, with *n* = 35 receiving placebo) found significantly less progression of cartilage damage by 3T MRI at 52 weeks, along with significant improvements in function and pain scores compared with placebo at 12, 52, and 72 weeks, using the International Knee Documentation Committee (IKDC) and visual analogue scale (VAS) scoring systems [99]. The improvement in IKDC scores was maintained at 104 weeks [99]. These results were supported by an independent phase II RCT in 86 patients, which also found reduced OA progression by MRI at 12 months [100]. Further trials in larger cohorts are required to ascertain whether these improvements in function and pain are maintained, or whether repeat administration of TGF-β1-expressing cells is required, with potential increased risk of fibrosis and osteophyte formation. A phase III trial is currently underway (NCT03203330). Strategies such as co-administration of SMAD7, which has been shown to block synovial fibrosis while enabling anabolic TGF-β1 signaling in chondrocytes [101], may be beneficial. 

### 3.5. Considerations around Receptor Expression and Downstream Signaling 

Many of the growth factors discussed here can signal through more than one cell surface receptor, leading to potentially disparate downstream biological effects. It is thus essential to understand receptor expression patterns and signaling pathways if growth factors are to be used therapeutically. 

For example, there are 4 FGF receptors (FGFRs), of which FGFR1 and FGFR3 are thought to be most important in cartilage. Some studies have concluded that FGF2 has catabolic effects on chondrocytes, inhibiting proteoglycan accumulation and aggrecan expression in vitro and ex vivo and inducing MMP-13 expression [102,103,104]. Other studies have ascribed a protective role to FGF2, with *Fgf2*^−/−^ mice developing accelerated spontaneous and surgically-induced OA, which could be reversed by addition of exogenous FGF2 [105]. Subsequent studies in human cartilage explants indicated that FGF2 could suppress IL-1-induced aggrecanase activity [106]. An elegant explanation for this disparity was put forward by Yan et al., who showed that the effects of FGF2 on the joint depended on which FGFR was activated, with the catabolic effects of FGF2 resulting from signaling through FGFR1 and not FGFR3 [107]. In OA, FGF2 is thought to shift towards catabolic signaling, with increased activation of FGFR1 relative to FGFR3. The molecular mechanism for this is yet to be elucidated, but may involve changes in relative FGFR expression or alteration in sulfation of heparan sulfate (HS) [108,109] that could alter formation and/or stability of trimolecular FGF2:FGFR:HS signaling complexes. FGF18 preferentially signals through FGFR3 [107], favoring anabolic rather than catabolic signaling. 

Similarly, TGF-β1 signaling through its type 1 receptor activin receptor-like kinase 5 (ALK5) leads to phosphorylation of SMAD3, inducing transcription of SMAD3-target genes such as *COL2* and *ACAN* [110], while signaling through ALK1 leads to phosphorylation of SMAD1/5/8 and transcription of catabolic genes such as *COLX* and *MMP13* [111]. ALK5 expression levels decrease with age, and the ALK1/ALK5 ratio is elevated in both OA and healthy aged cartilage [111,112], potentially favoring catabolic gene expression upon ageing. 

## 4. Targeting Therapies to Cartilage 

### 4.1. Strategies for Delivery to the Cartilage Matrix or to Chondrocytes

In addition to inhibiting cartilage loss and stimulating cartilage repair, a successful DMOAD must be able to reach its molecular target in cartilage and be retained at effective levels in the tissue. The negatively-charged cartilage extracellular matrix acts as a barrier to entry of many molecules, especially those with lipophilic properties. Several groups have identified the potential of cartilage ECM to act as a reservoir rather than as a barrier to entry, and are developing targeting strategies that exploit the composition and high negative fixed charge density of the matrix to deliver and retain DMOADs in cartilage. 

Here, we discuss some of these strategies, with particular focus on peptide-based moieties that bind to abundant and selectively expressed cartilage ECM molecules such as type II collagen and aggrecan (Table 1). These can be combined with protein therapeutics by standard protein engineering techniques and/or dendrimers to improve cartilage targeting and half-life at the desired site of action (Table 2), while reducing systemic and off-target effects and toxicity. These approaches may also be of use in rheumatoid arthritis, where the significant levels of inflammation and angiogenesis further promote delivery of potential therapies to the inflamed joint [113].

#### 4.1.1. Targeting Type II Collagen

Type II collagen is selectively expressed in cartilage and has a low rate of turnover in adult cartilage, making it attractive for targeting potential DMOADs to cartilage. 

Rothenfluh et al. [114] used phage display of peptide libraries to select a peptide (with the sequence WYRGRL) that selectively binds to type II collagen. While cross-reactivity with type I collagen was not directly evaluated in this study, strong cartilage targeting was observed in mice in vivo after intra-articular injection [114]. For example, the signal from WYRGRL-targeted fluorescent nanoparticles was 72-fold higher after 48 h than the signal from nanoparticles bearing a scrambled peptide [114]. The WYRGRL peptide has subsequently been used to deliver other cargo to cartilage, including dexamethasone [115], pepstatin A [116], and an HA-binding peptide [117,118] (Table 1).

A recently-reported collagen-targeting strategy utilizes avimers, which are artificial binding proteins that can be engineered to bind with high affinity to target molecules [125]. The avimer scaffold is based on protein A domains found in various cell surface receptors, and in vitro exon shuffling and phage display of these sequences generates binding moieties with high affinity and in vivo stability [125]. Hulme et al. used an avimer phage display library to generate avimers which bind to type II collagen with high affinity, enabling retention in rat knees for a month after intra-articular injection [126]. Fusion of the avimer with IL-1Ra generated a construct that was able to block IL-1 activity in rat knee joints in vivo, even when administered a week before the IL-1 challenge [126]. 

Phage display has also been utilized to generate single-chain variable fragments (scFv) that bind to type II collagen which has been post-translationally modified by reactive oxygen species (ROS) [127]. These antibodies selectively bound to damaged rheumatoid and osteoarthritic but not normal murine cartilage [127], indicating they can selectively target areas of joint damage. Cartilage binding was retained after coupling to payloads such as an MMP-cleavable form of viral IL-10 [128] and soluble TNF receptor II [127]. The fragments also enabled in vivo imaging of murine OA cartilage damage, with significant increases in signal 8 weeks after surgical destabilization of the medial meniscus (DMM) [129]. However, the relatively large size of scFv fragments (~27 kDa) makes avimers (~4 kDa) and peptides (850 Da for WYRGRL) more attractive for construction of targeted DMOADs.

#### 4.1.2. Targeting Aggrecan

Aggrecan is another abundant cartilage ECM molecule, with a high fixed charge density due its many chondroitin and keratan sulfate moieties. This property has been exploited for cartilage targeting, through strategies that use electrostatic interactions to increase binding and retention of positively charged molecules in the cartilage ECM [130,131,132,133] (Table 2). Cationic carriers that have been evaluated for cartilage delivery include peptides such as RRRR(AARRR)_3_R [131] and proteins such as avidin [130]. Avidin-conjugated dexamethasone was found to inhibit IL-1-driven aggrecan breakdown in cartilage explant cultures more effectively than soluble dexamethasone [134], illustrating the potential of this approach. Heparin-binding domains, such as those found in growth factors including FGF18, are cationic at neutral pH, and are thus also well-suited for cartilage delivery. For example, the heparin-binding domain of heparin-binding epidermal growth factor (HB-EGF) has been used to increase retention of IGF-1 in cartilage in vivo, with increased therapeutic efficacy in a rat medial meniscal tear model of OA [124]. One caveat of cationic delivery strategies is that they must be designed to support weak, reversible interactions with the cartilage matrix, as a high net positive charge can favor tight binding that limits penetrability [131].

#### 4.1.3. Targeting Chondrocytes

While some potential DMOADs are designed to act on targets found in the cartilage ECM (e.g., secreted enzymes), others may have intracellular targets that require targeting to chondrocytes rather than to the cartilage matrix. A chondrocyte-binding peptide with the sequence DWRVIIPPRPSA was identified by screening phage display peptide libraries against rabbit cartilage pieces [121]. The exact molecular target of this peptide has not been reported, but it was found to bind more than a scrambled peptide to human and rabbit chondrocytes, and also bound more to chondrocytes than to synovial cells [121]. Confocal analysis indicated cellular uptake of the peptide, enabling successful delivery of a DNA vector to chondrocytes in vivo, driving expression of green fluorescent protein and luciferase [121]. 

### 4.2. Increasing Sophistication to Tailor Avidity and Enable DMOAD Latency

Polymers and dendrimers have several advantages for cartilage targeting, including potential for increased avidity (e.g., through substitution with multiple copies of a targeting moiety [116]) and in vivo imaging (e.g., through inclusion of a fluorphor [116,140] or gadolinium MRI contrast agent [141]). Here we discuss recent progress with multivalent dendrimer scaffolds such as 1,4,7,10-tetraazacyclododecane-1,4,7,10-tetraacetic acid amide (DOTAM) and polyamidoamine (PAMAM), with nanoparticles and polymers (e.g., poly(lactic-co-glycolic acid)(PLGA) recently reviewed elsewhere [57,142,143].

The DOTAM scaffold contains a multivalent tetrapodal core with flexible polyethylene linkers that can be decorated with targeting peptides and/or cationic groups to promote cartilage retention [116]. For example, the scaffold has been coupled to the type II collagen-targeting peptide WYRGRL to deliver the cathepsin D inhibitor pepstatin A to cartilage [116]. In vivo retention of this conjugate increased as the number of WYRGRL peptides attached was increased from 1 to 3 [116]. WYRGRL-derivatized DOTAM has also been combined with gadolinium and Cy5.5 to enable in vivo dual MRI/near-infrared imaging of OA cartilage in a pre-clinical rat meniscal tear model [141]. Clinical translation for human imaging would require further safety profiling. 

PANAM dendrimers are similarly multivalent, enabling a combination of desirable targeting and imaging moieties. They are additionally positively charged, favoring electrostatic binding to the negatively charged cartilage ECM. Derivatization with PEG can be used to shield this positive charge in a tuneable manner, enabling tight control of matrix binding affinity. Such PEG-PANAM dendrimers have been used to deliver IGF-1 in vivo, achieving a 10-fold increase in residence times in rat knee joints and improved chondroprotection after surgical induction of OA [144]. Derivatization of PANAM dendrimers with targeting peptides such as the DWRVIIPPRPSA chondrocyte affinity peptide [121,145] may further improve their targeting efficacy. 

Another approach to cartilage targeting has been to create prodrugs that should only be activated in areas of the body where metalloproteinase activity is high, such as in OA joints. For example, IFNβ has been linked to the latency-associated peptide (LAP) of TGF-β1 via the MMP-cleavage sequence PLGLWA [137]. Intramuscular gene delivery of this construct reduced joint swelling in the collagen-induced arthritis model [137]. Linking IFNβ and LAP via the ADAMTS-cleavable sequence DVQEFRGVTAVIR improved in vivo targeting and therapeutic efficacy in this model [138]. Similar constructs may also be useful for delivery of cargo in OA. For example, a LAP-TIMP-3 construct can be activated by protease activity in the synovial fluid of OA patients [139], and may be useful for blocking metalloproteinase-mediated OA cartilage degradation.

## 5. Broader Challenges in DMOAD Development 

In addition to target identification, there are a number of additional factors to consider for the development of a successful disease-modifying OA drug. 

A successful DMOAD must be able to penetrate the highly-charged cartilage matrix to reach its target site. The pore size of the collagen network is ~100 nm, with glycosaminoglycan chains on aggrecan spaced 2–4 nm apart. Molecules up to 16 nm in diameter and 500 kDa in size have been shown to penetrate the cartilage matrix (reviewed in [133,146]), but entry is highly dependent on molecular charge, as discussed in Section 4.1 (Strategies for delivery to the cartilage matrix or to chondrocytes). As OA worsens, the charge and porosity of the cartilage matrix changes progressively, with consequent effects on DMOAD delivery and retention [119,147]. Targeting can be lost when matrix molecules are degraded, or, conversely, entry and retention can increase when cartilage is damaged. For example, cationic, collagen type II-targeted PLGA nanoparticles were found to accumulate more in OA than in normal cartilage in a rat collagenase-induced OA model, at a stage when proteoglycan loss was observed in 25–50% of the cartilage matrix [119]. Cartilage damage in early and mid-stage OA may thus promote DMOAD entry and retention, although the window of therapeutic opportunity is likely to close as cartilage damage progresses. 

The route of DMOAD administration is also a critical consideration. While many, but not all, molecules can pass from the blood stream into synovial fluid, the avascular nature of cartilage makes delivery and pharmacokinetics difficult to predict. DMOAD RCTs thus generally utilize intra-articular injection, although this has associated clinical risks and does not substantially increase the half-life of non-targeted DMOADs, as solutes are cleared from the joint space within 1–5 h of intra-articular injection [148]. Administration routes may have to alter if targeting strategies are introduced, depending on their molecular properties and mechanism of action.

Molecular mechanisms driving cartilage degradation and repair appear to be largely conserved in human and murine OA [149], but the substantial difference between cartilage thickness in the two species means that mice are unlikely to be a good model for evaluating targeting strategies such as those discussed here. For example, the half-life of fluorescently labeled avidin is 5–6-times shorter in rat than in rabbit cartilage [135]. 

One of the greatest obstacles in DMOAD development is the lack of a robust and rapid outcome measure for therapeutic efficacy, with joint space width measurements lacking sensitivity and specificity. Improved measures of OA progression, such as accurate biomarkers [150] and novel imaging modalities [151], will greatly assist in DMOAD development and are the focus of considerable attention in the field. 

Another key issue is whether OA pain correlates with structural joint changes [152]. For example, in a recent longitudinal study of 600 participants, Felson and colleagues [153] demonstrated that cartilage loss over 24 months correlated significantly with worsening of WOMAC knee pain over 24 and 36 months, but found that the effect size was small. This not only presents a challenge for RCT design, but calls into question whether successful DMOADs can be developed based on chondroprotection alone. Clinical trials of sprifermin, for example, have shown a significant effect on cartilage thickness, but not on pain [75,76]. Analysis is complicated by the strong placebo effect of intra-articular injection on patient-reported pain outcome measures [154,155], which has been observed in trials for sprifermin [75] and SM04690 [86], and by variations in scoring systems used to measure pain and in their practical application [156,157]. Promisingly, preclinical studies indicate that ADAMTS-5 inhibitors may have an analgesic effect [158], as aggrecan degradation by ADAMTS-5 generates a peptide that promotes OA pain via toll-like receptor 2 activation [159]. 

## 6. Conclusions

DMOAD development remains an important and challenging area, with substantial research effort focused on inhibiting metalloproteinase-mediated cartilage degradation and on promoting cartilage repair. A number of potential therapies have recently progressed to clinical trial, indicating that investments in fundamental research in the area are bearing fruit and delivering strong targets for drug development. Strategies that target these potential DMOADs to cartilage may help overcome the challenges of OA drug delivery, by utilizing the cartilage matrix as a drug reservoir while reducing potential systemic toxicity. This is of particular importance given the chronic nature of OA and the high prevalence of co-morbidities. 

## Figures and Tables

**Table 1:** Peptides tested for targeting of potential osteoarthritis (OA) therapies to cartilage.

Peptide	Identified by	Binds to	Delivers	In Vivo Efficacy	Delivery
WYRGRL [114]	Screening of phage display peptide libraries against collagenase D-treated bovine cartilage pieces	Type II collagen	Nanoparticles [114,119]; dexamethasone [115]; pepstatin A via DOTAM scaffold [116]; HA-binding peptide [117,118]	72-fold higher cartilage targeting compared with scrambled peptide [114]; 14-fold more retention of DOTAM-pepstatin A in murine knee joints, with ex vivo reduction in cathepsin D activity [116]	Intra-articular [114,116,118]
HSNGLPL [120]	Screening of phage display peptide libraries against TGFβ1	TGFβ1	TGFβ1 [120]	Increases cartilage regeneration in rabbit full thickness defect model [120]	Intra-articular during surgery to create cartilage defect [120]
DWRVIIPPRPSA [121]	Screening of phage display peptide libraries against rabbit cartilage pieces	Chondrocytes	DNA vector [121]; siRNA targeting *Hif2a* [122]	Higher uptake by chondrocytes than scrambled peptide [121]. Reduced cartilage damage in murine OA model than scrambled peptide [122]	Intra-articular [122]
RLDPTSYLRTFW and HDSQLEALIKFM [123]	Screening of phage display peptide libraries against cultured chondrocytes	Chondrocytes, at least in part via binding to aggrecan			
KRKKKGKGLGKKRDPSLRKYK [124]	Sequence taken from heparin-binding domain of HB-EGF [124]	Heparin in vitro, binding to HS in vivo not shown [124]	Fusion protein consisting of IGF-1 fused with a heparin-binding domain [124]	Increased in retention of IGF-1 and proteoglycan synthesis in cartilage in vivo. Reduced cartilage damage in rat knee OA model [124]	Intra-articular [124]

**Table 2:** Examples of scaffolds used for targeting of potential OA therapies to cartilage.

Strategy	Identified by	Binds to	Delivers	In Vivo Efficacy	Delivery
Cationic carriers (avidin, peptides, etc.) [130,131,132,134]	Various	Negatively-charged cartilage matrix	Dexamethasone [134]	In vitro: Improved retention of cargo in cartilage explants [134]	Intra-articular [135]
scFv	Screening of scFv phage display library against ROS-modified type II collagen [127]	ROS-modified type II collagen [127]	MMP-cleavable form of viral IL-10 [128]; soluble TNF receptor II [127]; anti-inflammatory extracellular vesicles [136]	Reduced inflammation in RA models [127,128]; in vivo imaging of murine OA [129]	Intra-peritoneal [127,128]; intravenous [136]
Avimer [126]	Screening of avimer phage display library against rat and human type II collagen	Type II collagen	IL-1Ra [126]	Blocked IL-1 activity in rat knee joints when administered at same time as IL-1, and also when administered 1 week before	Intra-articular [126]
Metalloproteinase-activatable prodrugs	Use of latency-associated peptide of TGFβ1 [137]	Cleaved by activating MMPs and ADAMTSs	IFNβ [137,138]; TIMP-3 [139]	Reduced joint swelling [137], in vivo targeting, and therapeutic efficacy in CIA model [138]	Intramuscular [136], intraperitoneal [138]
